# Supplementary material for: Nitrogen-Fixing Paenibacillus haidiansis and Paenibacillus sanfengchensis: Two Novel Species from Plant Rhizospheres
Source: Microorganisms. 2024 Dec 12;12(12):2561. doi: 10.3390/microorganisms12122561 (PMC11676665; doi:10.3390/microorganisms12122561)
Supplement: Supplementary file 1 [file microorganisms-12-02561-s001.zip › microorganisms-3275559-supplementary.pdf]

**A**

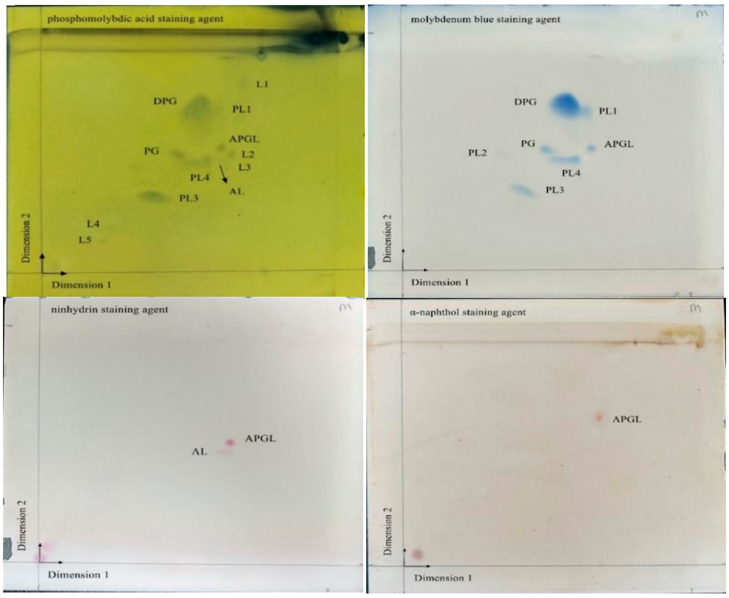


**B**

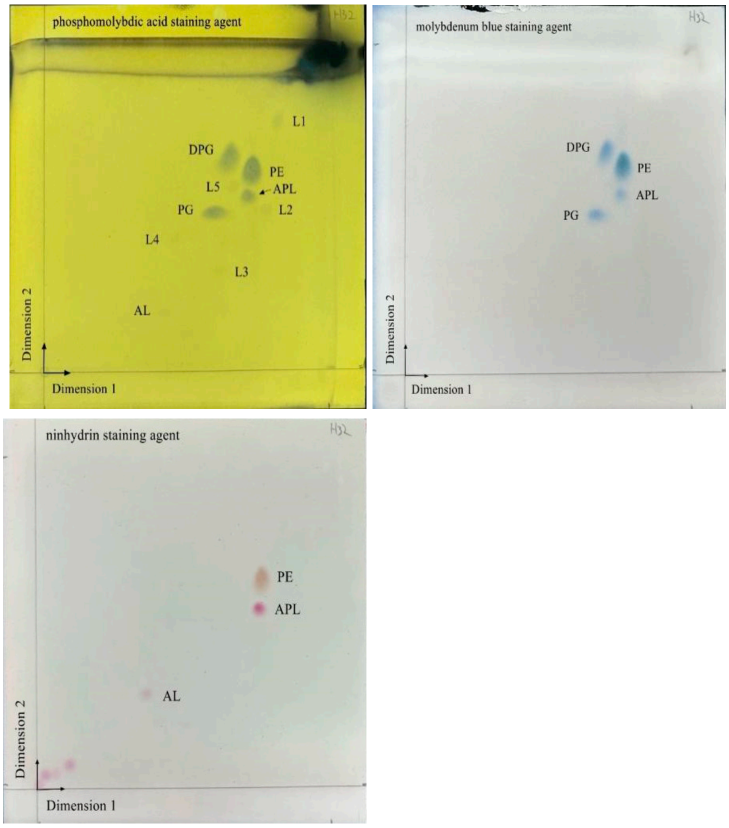


**Figure S1**. Two-dimensional TLC plate of polar lipids extracted from strain M1 (**A**) and from strain H32 (**B**). The plate was sprayed with 10% (v/v) molybdophosphoric aicd to show all polar lipids. DPG, diphosphatidylglycerol; PG, phosphatidylglycerol; PE, phosphatidylethanolamine; APL, aminophospholipids; AL, aminolipid; PL, unidentified phosphoglycolipids; L, unknown polar lipids; APGL, unidentified aminophosphoglycolipid.

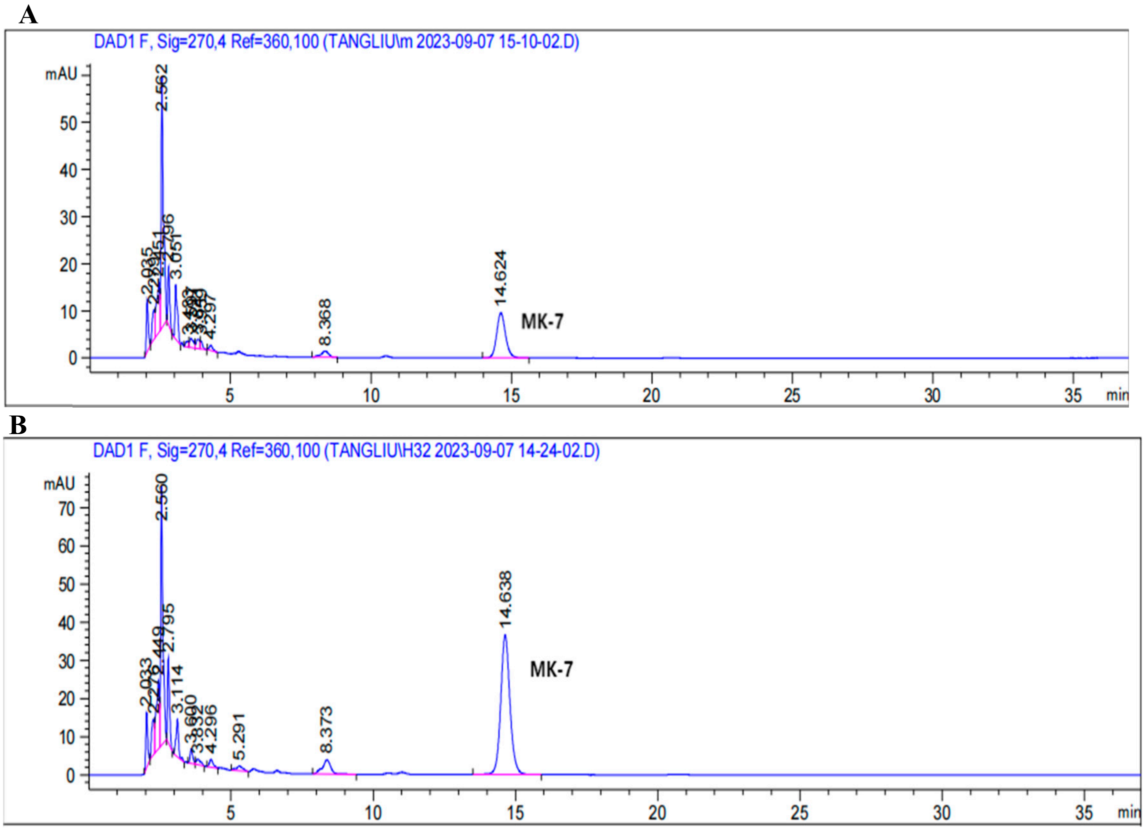


**Figure S2.** HPLC analysis shows MK-7 as the major respiratory quinone component for strain M1 (**A**) and for strain H32 (**B**). Mobile phase, methanol: isopropanol=65:35; Chromatographic column: Zorbax Eclipse XDB-C18 (4.6 * 250 mm, 5 µ m; Agilent); Column temperature: 40 ℃; Flow rate: 1.0 mL/min; Injection volume: 10 µ L; Detection wavelength: 270 nm.
